# Precipitation of Calcium, Magnesium, Strontium and Barium in Tissues of Four *Acacia* Species (Leguminosae: Mimosoideae)

**DOI:** 10.1371/journal.pone.0041563

**Published:** 2012-07-25

**Authors:** Honghua He, Timothy M. Bleby, Erik J. Veneklaas, Hans Lambers, John Kuo

**Affiliations:** 1 School of Plant Biology, The University of Western Australia, Crawley, Australia; 2 Centre for Microscopy, Characterisation & Analysis, The University of Western Australia, Crawley, Australia; Centre National de la Recherche Scientifique, France

## Abstract

Precipitation of calcium in plants is common. There are abundant studies on the uptake and content of magnesium, strontium and barium, which have similar chemical properties to calcium, in comparison with those of calcium in plants, but studies on co-precipitation of these elements with calcium in plants are rare. In this study, we compared morphologies, distributional patterns, and elemental compositions of crystals in tissues of four *Acacia* species grown in the field as well as in the glasshouse. A comparison was also made of field-grown plants and glasshouse-grown plants, and of phyllodes of different ages for each species. Crystals of various morphologies and distributional patterns were observed in the four *Acacia* species studied. Magnesium, strontium and barium were precipitated together with calcium, mainly in phyllodes of the four *Acacia* species, and sometimes in branchlets and primary roots. These elements were most likely precipitated in forms of oxalate and sulfate in various tissues, including epidermis, mesophyll, parenchyma, sclerenchyma (fibre cells), pith, pith ray and cortex. In most cases, precipitation of calcium, magnesium, strontium and barium was biologically induced, and elements precipitated differed between soil types, plant species, and tissues within an individual plant; the precipitation was also related to tissue age. Formation of crystals containing these elements might play a role in regulating and detoxifying these elements in plants, and protecting the plants against herbivory.

## Introduction

Biomineralisation, the formation of minerals by living organisms, is a widespread biological phenomenon displayed by most forms of life on Earth, including plants [1]. Some proposed functions of biomineralisation in plants include bulk element (mainly calcium) regulation [2], detoxification of aluminium and heavy metals [3], mechanical support [4], protection against herbivory [5], and light regulation during photosynthesis [6].

Calcium (Ca), an alkaline earth element, is the predominant cation associated with biomineralisation for most organisms, and calcium-bearing minerals comprise about 50% of known biominerals [1]. Calcium is an essential plant macronutrient and signalling compound, and it performs many fundamental functions in cellular metabolism [7], [8]. The formation of calcium crystals is common throughout the plant kingdom, and the most widely reported crystal is calcium oxalate [9].

Like calcium, magnesium (Mg) is an essential plant macronutrient; it is required for chlorophyll synthesis, ion transport and cation balance regulation, and as an activator of >300 enzymes [10]. Strontium and barium, on the other hand, have similar chemical properties to calcium [11], [12], but neither is essential for plant growth. However, both of these elements may be present in the soil in substantial amounts and have the potential to influence plant growth directly or indirectly through competition with and replacement of essential elements, i.e. calcium and magnesium [13]. Although there are numerous studies on the uptake and concentration of magnesium, strontium and barium together with those of calcium [14], [15], studies on co-precipitation of these elements with calcium in biogenic crystals are rare [16], [17], [18].

*Acacia* (Leguminosae: Mimosoideae) is a large and diverse genus, which is dominant in the vegetation of arid Australia [19], and plays an important role in nutrient, water and carbon cycling in arid ecosystems [20], [21]. Many Australian *Acacia* species are adapted to a range of habitats and soil types [22] and are preferred for land restoration after mining [23]. Biomineralisation is likely to contribute to detoxification of metals in *Acacia* plants grown in substrates with elevated metal levels, and studying biomineralisation may provide valuable information to select suitable plant species for growth on disturbed land, for example, mined soil that is calcium-rich.

In our previous study, an abundance of crystals containing calcium was observed in phyllodes (i.e. petioles functioning as leaves) of *Acacia robeorum*, and magnesium was co-precipitated with calcium [24]. Here, we expand our previous work and investigate crystal formation in four *Acacia* species (including *A. robeorum*) native to the Great Sandy Desert in north-western Australia. The aim of the present study was to assess: (1) whether there is any difference in crystal formation (including morphologies, distributional patterns, and elemental compositions) between species; (2) whether crystal formation differs between plants of the same species grown in different soil; (3) whether crystal formation is tissue-specific and related to tissue age. It is expected that results of this study, together with the results of our previous study [24], provide information on possible causes and potential functions of crystal formation in these *Acacia* species.

## Materials and Methods

### Plant Materials

The four *Acacia* species selected for the present study were *Acacia stipuligera* F. Muell., *Acacia ancistrocarpa* Maiden & Blakely, *Acacia stellaticeps* Kodela, Tindale & D. Keith, and *Acacia robeorum* Maslin. For these *Acacia* species, phyllodes are modified petioles that function as leaves [24], [25]. Samples were collected from mature plants grown in their natural habitat in the Great Sandy Desert in north-western Australia and from seedlings grown in a glasshouse for two years. Detailed descriptions of the study species and the Great Sandy Desert site are described in He et al. [20]. Healthy branches without any visible physical damage or disease were collected from the field. A coolbox half-filled with ice was used to store the fresh plant samples before they were transported to the laboratory for further preparation. All necessary permits were obtained for the described field studies. The study sites were unallocated crown land, which is managed by the Department of Environment and Conservation, Western Australia. A License to Take Flora for Scientific or Other Prescribed Purposes was issued by the Department of Environment and Conservation, Western Australia to collect the samples.

Seedlings of the four species were grown from seeds in 10-litre steam-sterilised potting mix for two years, and the free-draining pots were watered to their water-holding capacity with deionised water every three days. The bulk ingredients of the potting mix were fine composted pine bark, coco peat and river sand with a ratio of 5∶2:3 [v:v:v]. Additional ingredients in one litre of the potting mix included 1 g superphosphate [Ca(H_2_PO_4_)_2_], 0.3 g potassium sulfate (K_2_SO_4_), 1 g ammonium nitrate (NH_4_NO_3_), 0.5 g ferrous sulfate heptahydrate (FeSO_4_.7H_2_O), 2 g extra fine limestone (CaCO_3_), 2 g dolomite [(CaMg)(CO_3_)_2_], and 0.2 g macromin trace elements (Richgro, Perth, WA, Australia). There were three seedlings per species; however, all seedlings of *A. stipuligera* died, so only seedlings of the remaining three species were studied. For the three species, young (still expanding), mature (the youngest fully expanded) and old (the oldest healthy) phyllodes were collected for analysis along with branchlets, and lignified primary roots.

### Optical Microscopy

Optical microscopy was carried out on fresh, healthy and intact mature phyllodes collected from plants in the field. Samples were fixed in glutaraldehyde, infiltrated and embedded with glycol methacrylate (GMA) per He et al. [24]. Cross sections of embedded samples were cut with dry glass knives at a thickness of 2 µm, and sections were stained by the periodic acid-Schiff’s (PAS) reaction.

Images of the PAS-stained sections were captured under transmitted light with a Zeiss Axioplan Microscope equipped with a Zeiss Axiocam digital camera (Carl Zeiss, Oberkochen, Germany). Similar areas from unstained adjacent sections were imaged with an Olympus BX43 upright microscope (Olympus Corporation, Tokyo, Japan) equipped with crossed polarisers between which birefringent crystals appear as very bright objects against background darker tissue [26]. Due to rich cellulose deposits, xylem and fibrous cells also become birefringent [27].

### Scanning Electron Microscopy (SEM) and Energy-dispersive X-ray Spectroscopy (EDS)

Phyllodes from mature plants grown in the field and seedlings grown in the glasshouse were either cut with a double-edged razor blade or fractured by hand, while branchlets and primary roots were cut with a double-edged razor blade. All were fixed in formalin-acetic acid-alcohol (5% [v/v] formalin, 5% [v/v] acetic acid, and 70% [v/v] ethanol) (FAA). Calcium salts of phosphate and carbonate (if there were any) were removed by acetic acid [28], [29]. The FAA-fixed samples were dehydrated, critical point-dried, mounted and coated with gold for SEM imaging and X-ray microanalyses per He et al. [24]. Images were captured with a Zeiss 1555 VP-FESEM (Carl Zeiss) at 5–10 kV. Qualitative X-ray microanalyses were performed at 16 mm working distance on selected crystals using a Si (LI) EDS (Oxford Instruments, Oxford, England) on the same SEM at 20 kV.

All micrographs and spectra were processed using Photoshop CS5 software (San Jose, CA, USA), and processions included reproducing scale bars, cropping and aligning images.

## Results

### Morphologies and Locations of Crystals in Mature Plants Grown in the Field

**Figure 1:**
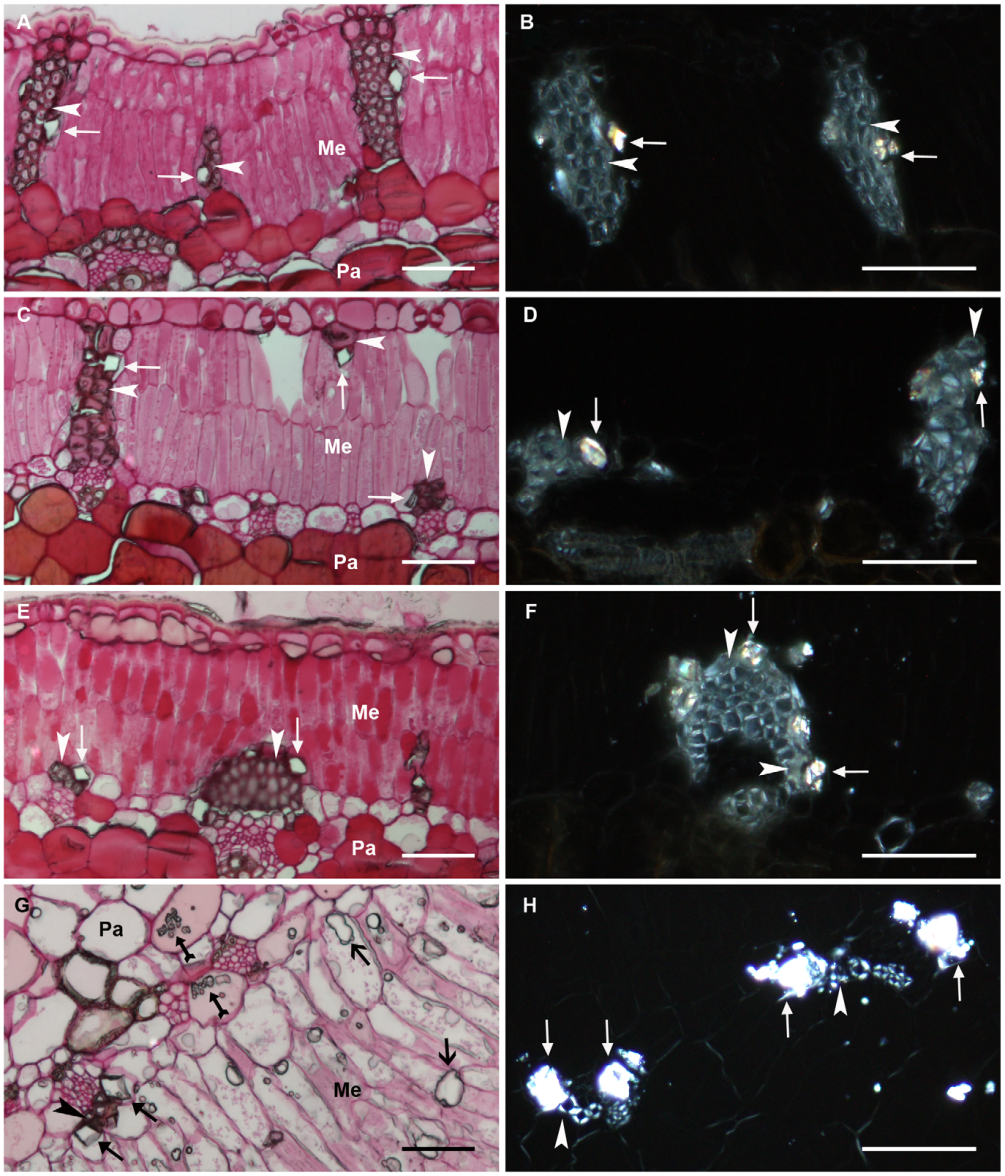
Optical microscopy images of cross sections of mature phyllodes collected from mature *Acacia* plants grown in the field. Filled arrow – prismatic crystal; filled arrow with tail – crystal sand; unfilled arrow – druses or spherical crystals; arrow head – fibre cell; Me – mesophyll; Pa – parenchyma. (A–B) *A. stipuligera*; (C–D) *A. ancistrocarpa*; (E–F) *A. stellaticeps*; (G–H) *A. robeorum*. (A), (C), (E) and (G) are views of sections stained by PAS reaction under transmitted light showing unstained crystals; (B), (D), (F) and (H) are polarised views of unstained sections showing birefringent crystals associated with fibre cells. All scale bars equal 50 µm.

Prismatic crystals were observed in cells associated with fibre cells in phyllodes of all four species collected from mature plants grown in the field, and all prismatic crystals were birefringent (Fig. 1). There were also other forms of crystals in phyllodes of *A. robeorum*, thought to be crystal sand, druses or spherical crystals (Fig. 1G), but these were not birefringent (Fig. 1H).

**Figure 2:**
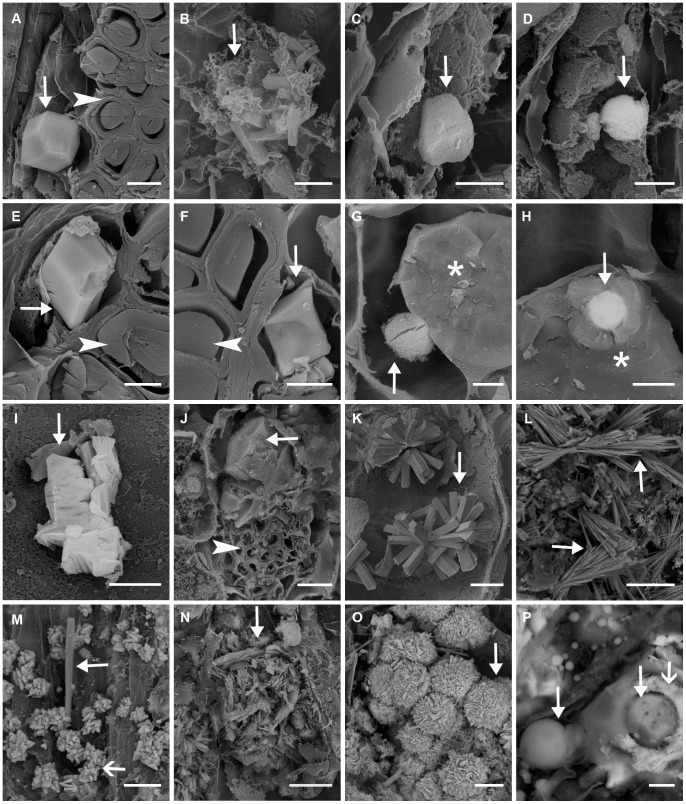
Scanning electron microscopy images of various crystals in mature phyllodes collected from mature *Acacia* plants grown in the field. Arrow – crystal; arrow head – fibre cell; asterisk – tannin deposit. (A) A prismatic crystal in a cell associated with fibre cells of *A. stipuligera*; (B) Styloids and styloid druses in the mesophyll of *A. stipuligera*; (C) A blocky crystal in a mesophyll cell of *A. stipuligera*; (D) A spherical crystal in a mesophyll cell of *A. stipuligera*; (E) A prismatic crystal in a cell associated with fibre cells of *A. ancistrocarpa*; (F) A prismatic crystal in a cell associated with fibre cells of *A. stellaticeps*; (G) A spherical crystal associated with tannin deposit in a parenchyma cell of *A. stellaticeps*; (H) A spherical crystal embedded in tannin deposit in a parenchyma cell of *A. stellaticeps*; (I) Amorphous crystals in a parenchyma cell of *A. stellaticeps*; (J) Prismatic crystals in cells associated with fibre cells of *A. robeorum*; (K) Styloid druses in a parenchyma cell of *A. robeorum*; (L) Raphides in the mesophyll of *A. robeorum*; (M) A styloid (filled arrow) and many druses (unfilled arrow) in the mesophyll of *A. robeorum*; (N) A cluster of platy aggregations in a mesophyll cell of *A. robeorum*; (O) Druses in a parenchyma cell of *A. robeorum*; (P) Spherical crystals (filled arrow) in mesophyll cells of *A. robeorum* including an amorphous aggregation (unfilled arrow) around a spherical crystal. Scale bars: (A, E, F, G, P) –5 µm; (B, C, D, H, I) –2 µm; (J, K, M, O) –10 µm; (L) –50 µm; (N) –20 µm.

Crystals of various morphologies were identified in different locations in phyllodes of field-collected plants (Fig. 2). For *A. stipuligera*, there were prismatic crystals in cells associated with fibre cells (Fig. 2A); styloids and druses (Fig. 2B), blocky crystals (Fig. 2C) and spherical crystals (Fig. 2D) were observed in mesophyll cells; and spherical crystals similar to the one shown in Fig. 2D were found in parenchyma cells (image not shown). Only prismatic crystals in cells associated with fibre cells were observed for *A. ancistrocarpa* (Fig. 2E). For *A. stellaticeps*, prismatic crystals in cells associated with fibre cells (Fig. 2F), spherical crystals associated with tannin deposits in parenchyma cells (Fig. 2G,H), and amorphous crystals in parenchyma cells (Fig. 2I) were observed. In phyllodes of *A. robeorum*, there were prismatic crystals in cells associated with fibre cells (Fig. 2J); styloid druses (Fig. 2K), raphides (Fig. 2L), styloids and druses (Fig. 2M) were present in both mesophyll and parenchyma cells; platy aggregation clusters were only observed in mesophyll cells (Fig. 2N). For *A. robeorum*, druses as shown in Fig. 2O were only found in parenchyma cells; spherical crystals as shown in Fig. 2P were present in both mesophyll and parenchyma cells, but amorphous aggregations around the spherical crystals were only found in mesophyll cells.

**Figure 3:**
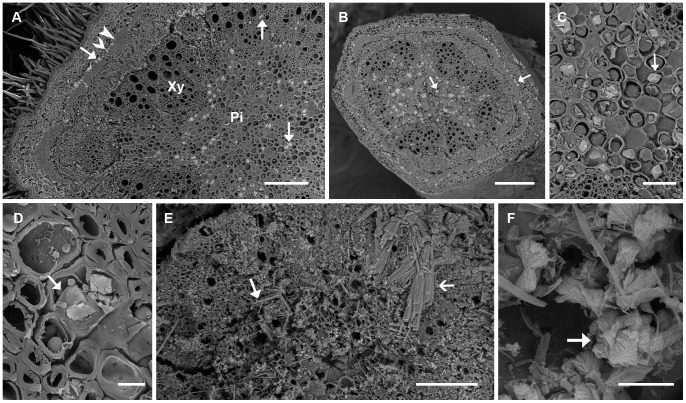
Scanning electron microscopy images of various crystals in branchlets collected from mature *Acacia* plants grown in the field. Arrow – crystal; filled arrow head – fibre cell; unfilled arrow head – cortical parenchyma cell; Pi – pith; Xy – xylem. (A) Part of a cross section of an *A. stipuligera* branchlet showing crystals mainly in pith, pith ray cells, xylem fibre cells, and in cortical parenchyma cells associated with phloem fibre cells; (B) A view of a whole cross section of an *A. ancistrocarpa* branchlet showing crystals mainly in pith, pith ray cells, xylem fibre cells and in cortical parenchyma cells associated with phloem fibre cells; (C) Prismatic crystals in pith of *A. ancistrocarpa*; (D) Crystals in pith ray cells of *A. stellaticeps*; (E) Solitary raphides (filled arrow) and a bundle of (pseudo-) raphides (unfilled arrow) in a branchlet of *A. robeorum*; (F) Bladed aggregation clusters in a branchlet of *A. robeorum*. Scale bars: (A, B) –200 µm; (C) –50 µm; (D) –10 µm; (E) –100 µm; (F) –5 µm.

Crystals were also formed in branchlets of field-collected plants of all four species (Fig. 3). For all four species, prismatic crystals were observed in pith, pith ray cells, xylem fibre cells and cortical parenchyma cells associated with phloem fibre cells (Fig. 3A-D). In addition, for *A. robeorum*, there were also solitary raphides and bundles of (pseudo-) raphides in all the above-mentioned tissues (Fig. 3E), and bladed aggregation clusters in xylem fibre cells (Fig. 3F). In phyllodes and branchlets of *A. robeorum*, there were more crystal types, as described in He et al. [24].

### Morphologies and Locations of Crystals in Seedlings Grown in the Glasshouse

**Figure 4:**
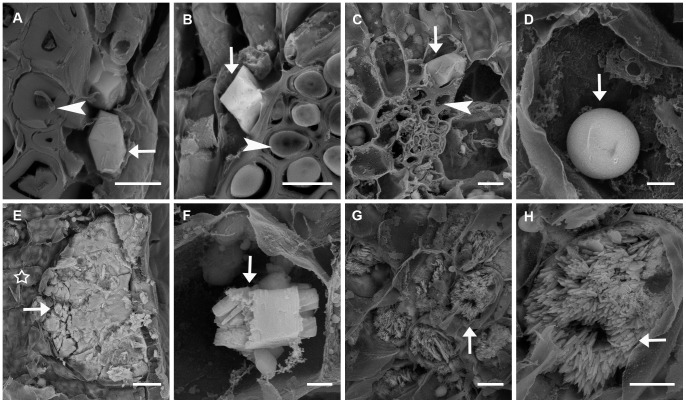
Scanning electron microscopy images of various crystals in phyllodes collected from *Acacia* seedlings grown in the glasshouse. Arrow – crystal; arrow head – fibre cell; pentagon – normal mesophyll cell. (A) Prismatic crystals in cells associated with fibre cells in a mature phyllode of *A. ancistrocarpa*; (B) A prismatic crystal in a cell associated with fibre cells in a mature phyllode of *A. stellaticeps*; (C) A prismatic crystal in a cell associated with fibre cells in a mature phyllode of *A. robeorum*; (D) A spherical crystal in a mesophyll cell in a mature phyllode of *A. robeorum*; (E) A crystal idioblast which is considerably larger than normal mesophyll cells in a mature phyllode of *A. robeorum*; (F) A crystal aggregation in a mesophyll cell in a mature phyllode of *A. robeorum*; (G) Bladed crystal aggregations in mesophyll cells in an old phyllode of *A. robeorum*; (H) An enlarged view of bladed crystal aggregations in (G). Scale bars: (A, B, C, G) –10 µm; (D, F) –2 µm; (e) –20 µm; (H) –5 µm.

In phyllodes collected from seedlings of *A. ancistrocarpa* and *A. stellaticeps*, there were only prismatic crystals in cells associated with fibre cells (Fig. 4A,B), and phyllodes of all ages (young, mature and old) showed the same pattern. However, for *A. robeorum*, the morphologies of crystals in phyllodes were more diverse. In *A. robeorum* phyllodes of all ages, in addition to prismatic crystals in cells associated with fibre cells (Fig. 4C), spherical crystals (Fig. 4D) were observed in almost every mesophyll and parenchyma cell; quite a few crystal idioblasts, which were considerably larger than normal mesophyll cells and with amorphous crystals inside, were formed in the mesophyll (Fig. 4E); crystal aggregations (Fig. 4F) (sometimes druses) were present sporadically in mesophyll and parenchyma cells. However, bladed crystal aggregations were only found in mesophyll cells in old phyllodes of *A. robeorum* (Fig. 4G,H), and they were present in almost every mesophyll cell and occupied quite a large proportion of a single cell, indicating that formation of this kind of crystals was related to phyllode age; no raphides were observed, and styloids only occurred sporadically in the mesophyll (image not shown).

**Figure 5:**
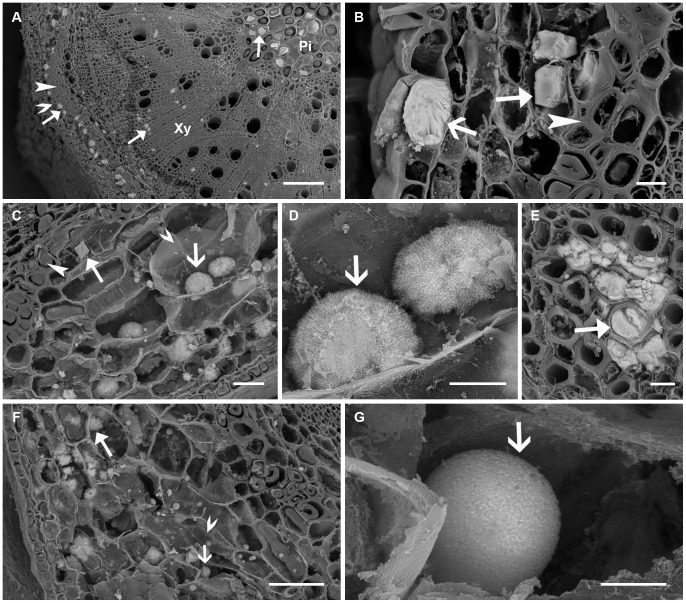
Scanning electron microscopy images of various crystals in branchlets collected from *Acacia* seedlings grown in the glasshouse. Arrow – crystal; filled arrow head – fibre cell; unfilled arrow head – cortical parenchyma cell; Pi – pith; Xy – xylem. (A) Part of a cross section of an *A. ancistrocarpa* branchlet showing crystals mainly in pith, xylem fibre cells, and in cortical parenchyma cells associated with phloem fibre cells; (B) Prismatic crystals (filled arrow) in cortical parenchyma cells associated with phloem fibre cells and an amorphous crystal (unfilled arrow) in an epidermal cell of *A. stellaticeps*; (C) A prismatic crystal (filled arrow) in a cortical parenchyma cell associated with phloem fibre cells and amorphous crystals (unfilled arrow) in cortical parenchyma cells of *A. robeorum*; (D) An enlarged view of two amorphous crystals in (C); (E) Amorphous crystals in xylem fibre cells of *A. robeorum*; (F) Amorphous (filled arrow) and spherical crystals (unfilled arrow) in cortical parenchyma cells of *A. robeorum*; (G) An enlarged view of a spherical crystal in (F). Scale bars: (A) –100 µm; (B, D, E) –10 µm; (C) –25 µm; (F) –50 µm; (G) –5 µm.

Similar to branchlets collected from mature plants grown in the field, branchlets collected from seedlings of *A. ancistrocarpa*, *A. stellaticeps* and *A. robeorum* all had prismatic crystals in pith, pith ray cells, xylem fibre cells and cortical parenchyma cells associated with phloem fibre cells (Fig. 5 A-C). Only prismatic crystals were observed in branchlets of *A. ancistrocarpa* (Fig. 5A). In addition to prismatic crystals, there were sporadic amorphous crystals in epidermal cells of *A. stellaticeps* branchlets (Fig. 5B); for branchlets of *A. robeorum*, there were also amorphous crystals in cortical parenchyma cells (Fig. 5C,D,F) and xylem fibre cells (Fig. 5E), and spherical crystals in cortical parenchyma cells (Fig. 5F,G).

**Figure 6:**
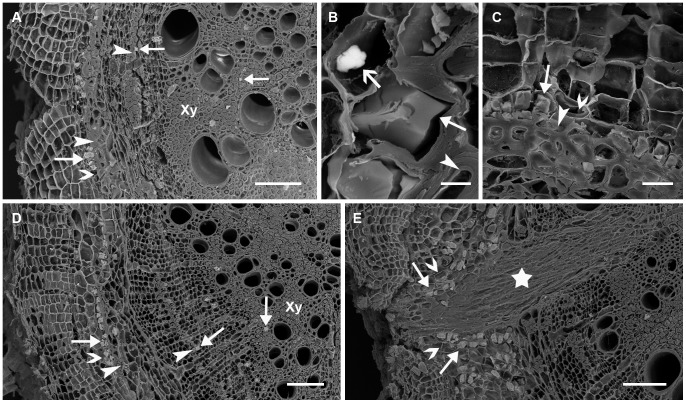
Scanning electron microscopy images of various crystals in primary roots collected from *Acacia* seedlings grown in the glasshouse. Arrow – crystal; filled arrow head – fibre cell; unfilled arrow head – cortical parenchyma cell; Xy – xylem: pentagon – developing lateral root. (A) Part of a cross section of an *A. ancistrocarpa* primary root showing crystals mainly in parenchyma cells associated with phloem fibre cells and in xylem fibre cells; (B) Prismatic crystals (filled arrow) in cortical parenchyma cells associated with phloem fibre cells and an amorphous crystal (unfilled arrow) in a cortical parenchyma cell of *A. ancistrocarpa*; (C) Prismatic crystals in cortical parenchyma cells associated with phloem fibre cells of *A. stellaticeps*; (D) Part of a cross section of a primary root of *A. robeorum* showing prismatic crystals mainly in parenchyma cells associated with phloem fibre cells and in xylem fibre cells; (E) Prismatic crystals in cortical parenchyma cells close to a developing lateral root of *A. robeorum*. Scale bars: (A, D, E) –100 µm; (B) –5 µm; (C) –20 µm.

Primary roots of *A. ancistrocarpa*, *A. stellaticeps* and *A. robeorum* all had prismatic crystals, and these crystals were mainly present in cortical parenchyma cells and vascular cambial cells associated with phloem fibre cells and in xylem fibre cells (Fig. 6A-E). In addition to prismatic crystals, there were sporadic amorphous crystals in cortical parenchyma cells in primary roots of *A. ancistrocarpa* (Fig. 6B); in contrast, only prismatic crystals were observed in primary roots of *A. stellaticeps* and *A. robeorum*, and it is noticed that almost every cortical parenchyma cell close to a developing lateral root had a prismatic crystal within it (Fig. 6E).

### Elemental Compositions of Various Crystals

**Figure 7:**
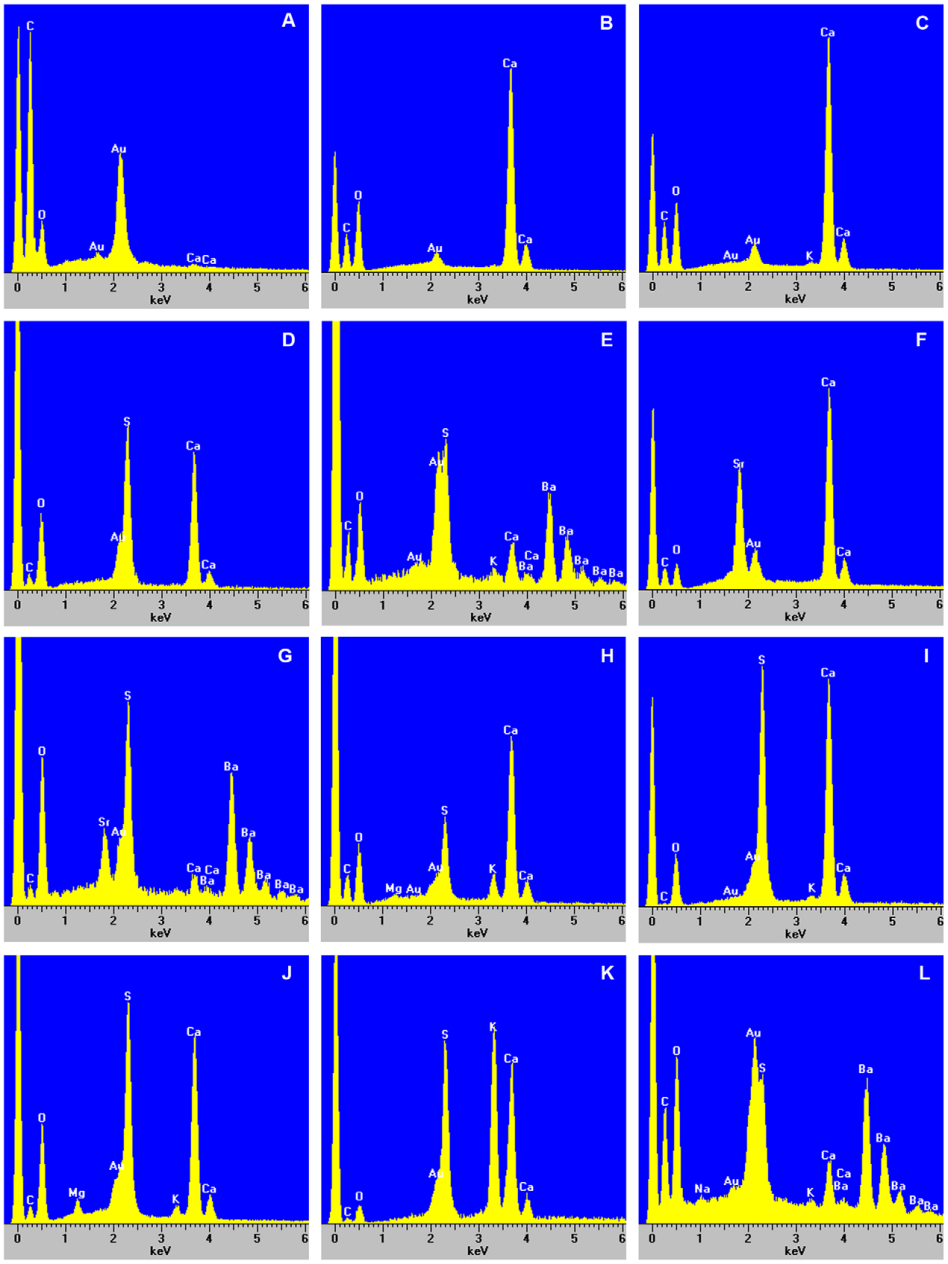
Typical spectra from energy-dispersive X-ray spectroscopy of various crystals in phyllodes, branchlets and roots of four *Acacia* species. For all spectra, the large peak to the left of the carbon (C) peak is background noise, and the peak of gold (Au) is from the gold used to coat the samples. (A) Typical spectrum showing only C, oxygen (O) and small Ca peaks from EDS of cells without crystals; (B–L) Typical spectra of various crystals. See Table 1 for a summary of these spectra and their corresponding crystal types.

Qualitative X-ray microanalyses showed that crystals in the four *Acacia* species were of various elemental compositions (Fig. 7, Table 1). Cells without crystals typically showed only carbon (C), oxygen (O), and small calcium (Ca) peaks (Fig. 7A); sometimes, spectra of cells without crystals showed no Ca peak (data not shown). For mature plants grown in the field, the most common type of crystal was presumably calcium oxalate, which showed large Ca, C and O peaks (Fig. 7B). Crystals of this composition included most prismatic crystals (except some in phyllodes of *A. stipuligera* and all in phyllodes and branchlets of *A. ancistrocarpa*), styloids, raphides, styloid druses, and some spherical crystals (Fig. 2G). For some prismatic crystals in phyllodes of field-grown *A. stipuligera*, there were small potassium (K) peaks (Fig. 7C).

**Table 1:** A summary of elements precipitated in various crystals in four *Acacia* species.

Spectrum	Elementsprecipitated	Crystal type and location	
		Field-grown plants	Glasshouse-grown plants
Fig. 7A	Ca, C, O (very small orno Ca peaks)	Cells without crystals in phyllodes, and branchlets of allfour species	Cells without crystals in phyllodes, branchlets, and primary roots of all three species
Fig. 7B	Ca, C, O (largeCa peaks)	Some prismatic crystals in cells associated with fibre cells inphyllodes of *A. stipuligera* (Fig. 2A); all prismatic crystalsin cells associated with fibre cells in phyllodes of*A. stellaticeps* (Fig. 2F, filled arrow) and *A. robeorum* (Fig. 2J,filled arrow); styloids and druses in mesophyll cells inphyllodes of *A. stipuligera* (Fig. 2B); spherical crystalsassociated with tannin deposits in parenchyma cellsin phyllodes of *A. stellaticeps* (Fig. 2G); styloid druses (Fig. 2K),raphides (Fig. 2L), styloids (Fig. 2M, filled arrow) in mesophylland parenchyma cells in phyllodes of *A. robeorum*; prismaticcrystals in pith, pith ray cells, xylem fibre cells and corticalparenchyma cells associated with phloem fibre cells inbranchlets of *A. stipuligera* and *A. stellaticeps* (Fig. 3A,D);solitary raphides and bundles of (pseudo-) raphides in pith,pith ray cells, xylem fibre cells and cortical parenchyma cellsassociated with phloem fibre cells in branchlets of *A. robeorum* (Fig. 3E).	All prismatic crystals in cells associated with fibre cells in phyllodes of all three species (Fig 4. A,B,C); all prismatic crystals in pith, pith ray cells, xylem fibre cells and cortical parenchyma cells associated with phloem fibre cells in branchlets of all species (Fig. 5A,B,C, filled arrows); all prismatic crystals in cortical parenchyma cells and vascular cambial cells associated with phloem fibre cells and in xylem fibre cells in primary roots of all three species (Fig. 6A-D, filled arrows).
Fig. 7C	Ca, K, C, O	Some prismatic crystals in cells associated with fibre cells inphyllodes of *A. stipuligera* (Fig. 2A).	
Fig. 7D	Ca, S, C, O	Blocky crystals in mesophyll cells of *A. stipuligera* (Fig. 2C);amorphous crystals in parenchyma cells of *A. stellaticeps*(Fig. 2I); druses (Fig. 2M, unfilled arrow) in mesophyll andparenchyma cells, platy aggregation clusters in mesophyllcells (Fig. 2N), druses in parenchyma cells (Fig. 2O), andamorphous aggregations around spherical crystals (Fig. 2P,unfilled arrow) in mesophyll cells in phyllodes of*A. robeorum*; bladed aggregation clusters in xylem fibrecells in branchlets of *A. robeorum* (Fig. 3F).	Amorphous crystals in crystal idioblasts in the mesophyll (Fig. 4E) and crystal aggregations in mesophyll and parenchyma cells (Fig. 4F) of *A. robeorum*.
Fig. 7E	Ca, Ba, K, S, C, O	Spherical crystals in mesophyll cells in phyllodes *A. stipuligera*(Fig. 2D).	
Fig. 7F	Ca, Sr, C, O	Prismatic crystals in cells associated with fibre cells in phyllodesof *A. ancistrocarpa* (Fig. 2E); prismatic crystals in pith, pithray cells, xylem fibre cells and cortical parenchyma cellsassociated with phloem fibre cells in branchlets of *A. ancistrocarpa* (Fig. 3B,C)	
Fig. 7G	Ca, Sr, Ba, S, C, O	Spherical crystals associated with tannin deposits inparenchyma cells in phyllodes of *A. stellaticeps* (Fig. 2H).	
Fig. 7H	Ca, Mg, K, S, C, O		Spherical crystals in mesophyll and parenchyma cells in phyllodes of *A. robeorum* (Fig. 4D); spherical crystals in cortical parenchyma cells in branchlets of *A. robeorum* [Fig. 5F (unfilled arrow), G].
Fig, 7I	Ca, K, S, C, O	Druses (Fig. 2M, unfilled arrow) in mesophyll and parenchymacells, platy aggregation clusters in mesophyll cells (Fig. 2N),druses in parenchyma cells (Fig. 2O), and amorphousaggregations around spherical crystals (Fig. 2P, unfilled arrow)in mesophyll cells in phyllodes of *A. robeorum*; bladedaggregation clusters in xylem fibre cells in branchlets of*A. robeorum* (Fig. 3F).	Amorphous crystals in crystal idioblasts in the mesophyll (Fig. 4E) and crystal aggregations in mesophyll and parenchyma cells (Fig. 4F) of *A. robeorum*.
Fig. 7J	Ca, Mg, K, S, C, O		Bladed crystal aggregations in mesophyll cells in old phyllodes of *A. robeorum* (Fig. 4G,H).
Fig. 7K	Ca, K, S, C, O		Amorphous crystals in epidermal cells of *A. stellaticeps* branchlets (Fig. 5B, unfilled arrow); amorphous crystals in cortical parenchyma cells [Fig. 5C (unfilled arrow),D,F (filled arrow)] and xylem fibre cells (Fig. 5E) of *A. robeorum*.
Fig. 7L	Ca, Ba, Na, K, S, C, O		Amorphous crystals in cortical parenchyma cells in primary roots of *A. ancistrocarpa* (Fig. 6B, unfilled arrow).

Some crystals showed large Ca, sulfur (S), O and small C peaks and were possibly mixtures of calcium sulfate and calcium oxalate with calcium sulfate being the major component (Fig. 7D); these crystals were observed in phyllodes of all species, except *A. ancistrocarpa*, and in branchlets of *A. robeorum*. In phyllodes of *A. stipuligera*, barium (Ba) and potassium (K) were precipitated in some crystals together with calcium, and these crystals were presumably mixtures of oxalate and sulfate salts of these elements (Fig. 7E). Prismatic crystals in phyllodes and branchlets of *A. ancistrocarpa* showed large Ca and strontium (Sr) peaks, indicating significant amount of strontium was incorporated into calcium oxalate crystals (Fig. 7F). Some crystals in phyllodes of *A. stellaticeps* showed large Sr, Ba, S and O peaks, while Ca and C peaks were small, and these crystals possibly contained sulfate and oxalate salts of calcium, strontium and barium, with barium sulfate being the major component (Fig. 7G). For elemental compositions of various crystals in phyllodes and branchlets of field-grown *A. robeorum*, see He et al. [24] for details.

For phyllodes, branchlets and primary roots from seedlings of *A. ancistrocarpa*, *A. stellaticeps* and *A. robeorum*, all prismatic crystals were calcium oxalate with only Ca, C and O peaks (Fig. 7B). For phyllodes and branchlets of *A. robeorum*, spherical crystals possibly contained sulfate and oxalate salts of calcium, magnesium and potassium (Fig. 7H). The amorphous crystals in mesophyll crystal idioblasts and crystal aggregations (or druses) mainly comprised calcium sulfate (Fig. 7D), and occasionally a small amount of potassium sulfate/oxalate (Fig. 7I). Bladed crystal aggregations were possibly mixtures of sulfate and oxalate salts of calcium, magnesium and potassium, with calcium sulfate being the major component (Fig. 7J). For amorphous crystals in epidermal cells of *A. stellaticeps* branchlets, and in cortical parenchyma cells and xylem fibre cells of *A. robeorum* branchlets, the large Ca, K, S peaks and small C peak indicated that both calcium sulfate and potassium sulfate were major components of the crystals (Fig. 7K). In primary roots of *A. ancistrocarpa*, in addition to calcium oxalate, a few amorphous crystals of which the elemental composition was complex were observed, and these crystals possibly contained oxalate and sulfate salts of calcium, barium, sodium and potassium (Fig. 7L).

## Discussion

Crystal formation differed between species, between plants of the same species grown in the field and in the glasshouse, and between tissues that differed in age. The morphologies, distributional patterns and elemental compositions of crystals in *A. robeorum* were the most diverse among the four *Acacia* species studied. Besides calcium, these crystals contained magnesium, strontium and barium. By comparing crystals in different tissues of mature plants grown in the field and seedlings grown in the glasshouse, possible causes of precipitation of these elements and potential functions of crystals containing these elements are discussed.

### Diversities in Crystal Morphology and Distribution

In this study of four *Acacia* species, an abundance of crystals representing almost all major crystal types that have been reported in the plant kingdom were found [9], [30]. Unlike some members of the Leguminosae family that contain crystals with very specific patterns of distribution [31], crystals were found in multiple tissues of an individual *Acacia* species, and the distribution of crystals within the plant varied between species. Among the four species studied, *A. robeorum* was a striking example of a species that formed numerous crystals of various morphologies in multiple tissues; see He et al. [24] for more information. These results challenge the traditional view that the morphologies and precise locations of crystals in plants are under strict genetic control, and that a particular species will form only a certain crystal type or subset of crystal morphologies [9].

### Possible Causes of Precipitation of Certain Elements

In addition to having diverse morphologies and distributional patterns, the elemental compositions of crystals observed in the four *Acacia* species also varied considerably. As expected, calcium was the dominant element involved in crystal formation, and calcium oxalate was the most common type of crystal formed, consistent with reports in the literature that calcium oxalate is widespread in all three subfamilies of the Leguminosae [31]. These results are further evidence that calcium precipitation is the most common form of biomineralisation in plants [9].

Precipitation of most elements may be biologically induced, rather than constitutive; that is, the secondary precipitation of minerals occurs as a result of interactions between biological activity and the environment [1]. As calcium is the most common cation in biomineralisation processes, and magnesium, strontium and barium follow calcium closely during soil-to-plant transfer [32], it is very likely that these elements will be precipitated in plants similarly to calcium. For calcium and magnesium, which are essential for plant growth, plants may not be able to reduce their uptake beyond the requirement for the plants’ metabolism, and the excess may be precipitated; for strontium and barium, which are not essential for plant growth and may actually be toxic to plants [33], precipitation might be a detoxification mechanism.

In the present study, when comparing crystals in mature plants grown in the field and seedlings grown in the glasshouse, differences in elemental compositions for the same species were noticed. Therefore, precipitation of calcium, magnesium, strontium and barium may be a response to the abundance of these elements in soil. All alkaline earth elements (calcium, magnesium, strontium and barium) are absorbed by plants in the ionic form; their quantities and ratios to each other vary considerably in different soils, and their availability to plants is affected by many soil and environmental factors [13], [32], [34]. In addition, calcium, magnesium, strontium and barium are chemically similar, they behave similarly during soil-to-plant transfer [32], [35], [36], and strontium is considered a good tracer for calcium distribution in citrus leaves [37]. Some plants show preferential uptake of certain elements while discriminating against others [11], [38], [39].

When comparing crystals in seedlings grown in the same soil in the glasshouse, it was found that the elements precipitated were different between species. Species vary in the uptake of alkaline earth elements from the same soil, partly due to differences in cation-exchange capacity of their roots [7], [40]. Among the four species, *A. robeorum* may have the highest cation-exchange capacity for calcium and magnesium, as reflected by numerous crystals containing calcium and magnesium, and its significantly higher foliar calcium and magnesium concentrations compared with those of the other three species [24].

Elemental compositions of crystals also differed between tissues of the same plants. For mature plants of *A. stipuligera* and *A. stellaticeps* grown in the field, precipitation of strontium and/or barium was observed in phyllodes, but not in branchlets; there might be a special mechanism involved in transporting them to phyllodes without having them precipitated in the xylem. Some organic ligands (e.g., anions of citrate and/or malate), which can form complexes with strontium and barium and increase the mobility and facilitate the upward movement of the two elements, may play an important role [15]. After arriving in vacuoles in phyllodes, the two elements were possibly dissociated from the organic complexes and precipitated with other organic and/or inorganic ligands to form more stable crystalline deposits (e.g., oxalate and/or sulfate). Elemental compositions of crystals in mature phyllodes and branchlets of *A. ancistrocarpa* grown in the field were similar; the calcium to strontium ratio may differ, but no quantitative analysis was attempted to confirm that. It is not clear why barium was precipitated in primary roots but not in phyllodes of seedlings of *A. ancistrocarpa* grown in the glasshouse. Precipitation of potassium in branchlets of *A. stellaticeps* and potassium and magnesium in branchlets of *A. robeorum* grown in the glasshouse warrants further study.

In most cases, crystals in plants have been identified as calcium oxalate [9], [29], and a few studies have reported the presence of calcium sulfate [41], [42], [43] and magnesium oxalate [44]. The present study shows that crystalline oxalate and sulfate salts of calcium, magnesium, strontium, and barium are formed in the studied *Acacia* plants. Precipitation of these elements with sulfate ions might occur due to ready access to sulfur from sulfur-rich substrate. Foliar sulfur concentrations of the four *Acacia* species (H. He, unpublished data), except *A. ancistrocarpa* in which no sulfate crystals were formed, were higher than the level considered adequate for growth of most plants (1 mg g^−1^ of plant dry mass) [45]. These *Acacia* species may prevent accumulation of alkaline earth metals in their rhizosphere by absorbing them in large quantities, and enhanced sulfur uptake might be induced to maintain homeostasis of these metals or charge balance in plant cells [46], [47].

Potassium is an essential nutrient and generally the most abundant cation in plant cells [48]. It is noticed that potassium was often precipitated, presumably in the form of potassium sulfate, and sometimes potassium sulfate could be the dominant component of some crystals found in branchlets of *A. stellaticeps* and *A. robeorum* seedlings grown in the glasshouse. The results of our previous study [24] showed that foliar potassium concentrations of mature plants of the four *Acacia* species grown in the field were well below the level required adequate for growth of most plants (10 mg g^−1^ of plant dry mass) [45]. The present *Acacia* species, which have inherently slow grow rates, may have a low potassium requirement, so potassium that was absorbed in excess of the plants’ requirement was precipitated. Precipitation of all elements could also be related to age of tissues, because the number of crystals in phyllodes and roots (data not shown) increased with age.

The accumulation and compartmentation of a certain element in plants appear to be species- and cell-specific, and the accumulation and compartmentation of different elements may vary in the same plant species or even in a single cell. It would be interesting to reveal mechanisms underlying the accumulation and compartmentation of various elements in plants. Studying expression of key ion or solute transporters under different growth conditions (including soil, water, temperature, herbivory), and at different plant developmental stages would partly explain the accumulation and compartmentation patterns of different elements [37], [47].

### Possible Functions of the Crystals

A number of functions have been proposed for the formation of calcium oxalate crystals in plants [9]. For the present *Acacia* species, the most likely function of the crystals containing calcium, magnesium, strontium and barium is regulating levels of these elements in metabolic compartments in the plants and avoiding toxicity [49], [50]. In addition, these crystals, depending on their shape, size, placement, abundance, and possibly elemental composition as well, may also prevent herbivory by large animals as well as insects [51], [52].

### Conclusions

In addition to magnesium, which was found to be co-precipiated with calcium in *A. robeorum* in our previous study, strontium and barium were precipitated together with calcium in the other three species. These elements were most likely precipitated in forms of oxalate and sulfate in plants. In most cases, precipitation of these elements was biologically induced, rather than constitutive, and precipitates differed between soil types, plant species, and tissues within an individual plant; the precipitation was also related to tissue age. Formation of crystals containing calcium, magnesium, strontium and barium may play a role in regulating metabolic concentrations and detoxifying these elements in plants, and protecting the plants against herbivory.
